# ^18^F-Fluorocholine PET/CT, Tc-99m-MIBI and TC-99m-MDP SPECT/CT in Tertiary Hyperparathyroidism with Renal Osteodystrophy

**DOI:** 10.3390/diagnostics10100851

**Published:** 2020-10-20

**Authors:** Cristina Ferrari, Valentina Lavelli, Giulia Santo, Maria Teresa Frugis, Francesca Iuele, Giuseppe Rubini, Angela Sardaro

**Affiliations:** 1 Nuclear Medicine Unit, Interdisciplinary Department of Medicine, University of Bari Aldo Moro, Piazza Giulio Cesare 11, 70124 Bari, Italy; valentina.lavelli@gmail.com (V.L.); giuliasanto92@gmail.com (G.S.); mariateresafrugis@hotmail.it (M.T.F.); francescaiuele@hotmail.com (F.I.); giuseppe.rubini@uniba.it (G.R.); 2 Section of Radiology and Radiation Oncology, Interdisciplinary Department of Medicine, University of Bari Aldo Moro, Piazza Giulio Cesare 11, 70124 Bari, Italy; angela.sardaro@uniba.it

**Keywords:** tertiary hyperparathyroidism, renal osteodystrophy, dual-phase Tc-99m-MIBI, ^18^F-fluorocholine, positron emission tomography/computed tomography, Tc-99m-MDP

## Abstract

Tertiary hyperparathyroidism (HPT) is a metabolic disorder characterized by the semi-autonomous hypersecretion of parathyroid hormone (PTH), leading to hypercalcemia. It can be the end result of persistent secondary hyperparathyroidism and is most commonly observed in patients with long-standing chronic kidney disease (CKD) and often after renal transplantation. Untreated HPT can lead to progressive bone disease, fibrocystic osteitis, and soft-tissue calcifications, along with other severe complications. In the 2009 Kidney Disease Improving Global Outcomes (KDIGO) guidelines, CKD-Mineral and Bone Disorder (CKD-MBD) is used to describe the broader clinical syndrome encompassing mineral, bone, and calcific cardiovascular abnormalities that develop as a complication of CKD. We report a 62-year-old female with a severe HPT evolved from advanced chronic kidney disease (stage 5D, KDIGO). Patient was evaluated with multimodality nuclear medicine functional imaging to assess hyperfunctioning parathyroid glands and bone lesions. Tc-99m-methoxyisobutylisonitrile (MIBI) dual-phase scintigraphy, Tc-99m-methylenediphosphonate (MDP) bone scan and ^18^F-Fluorocholine positron emission tomography/computed tomography (^18^F-FCH PET/CT) were performed before surgery.

**Figure 1:**
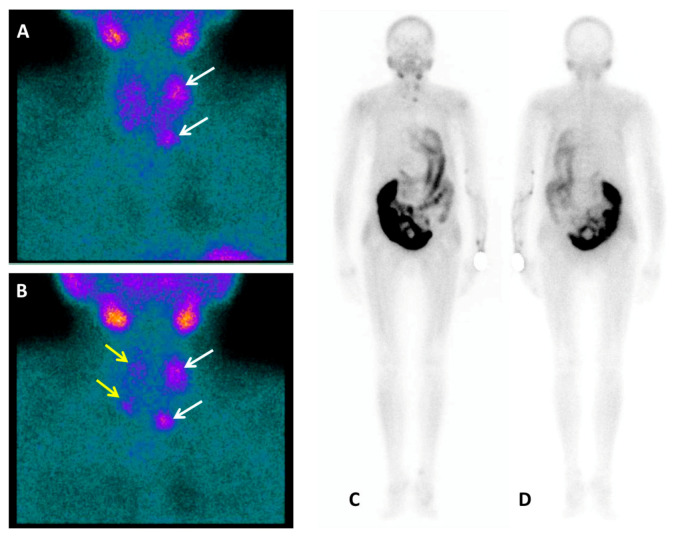
A nephrectomised 62-year-old woman with chronic renal failure (stage 5D, Kidney Disease Improving Global Outcomes (KDIGO)) [1] in dialysis treatment since 2012. Listed for kidney transplantation, laboratory tests were consistent with severe hyperparathyroidism: Parathyroid hormone (PTH) level 2068 pg/mL (normal range 15–57 pg/mL), total calcium level of 7.7 mg/dL (normal range 8.5–10.1 mg/dL), ionized calcium level of 3.49 mg/dL (normal range 4.6–5.3 mg/dL) and inorganic phosphate level of 3.7 mg/dL (normal range 2.5–4.9 mg/dL). Then, patient performed neck ultrasound (US) and, for the recent occurrence of dorsal back pain, a whole-spine magnetic resonance (MRI). According to tertiary hyperparathyroidism (HPT) diagnosis [2] the nuclear medicine imaging workup before surgery consisted of Tc-99m-methoxyisobutylisonitrile (Tc-99m-MIBI) and Tc-99m-methylenediphosphonate (Tc-99m-MDP) planar scintigraphy, completed with Single Photon Emission Computed Tomography/Computed Tomography (SPECT/CT), and ^18^F-FCH PET/CT. Tc-99m-MIBI parathyroid dual-phase planar scintigraphy (anterior view) showed on the early acquisition (**A**) a left superior hyperfunctioning parathyroid gland together with a left inferior one slightly under the lower third of the left thyroid lobe (white arrows). The delayed acquisition (**B**) demonstrated, after thyroid gland radiotracer washout, persistent uptake in left parathyroid glands and a mild Tc-99m-MIBI concentration also in the other two right glands (yellow arrows). Planar scintigraphy was integrated with a whole-body acquisition [3] because of the detection of lytic bone lesions on the whole-spine MRI, performed a few days before, doubtful for renal osteodystrophy, giant cell bone tumour, or carcinoma. No Tc-99m-MIBI uptake was found in the bone as showed in the whole body anterior (**C**) and posterior (**D**) planar views. Then, patient was directed to Tc-99m-MDP bone scan.

**Figure 2:**
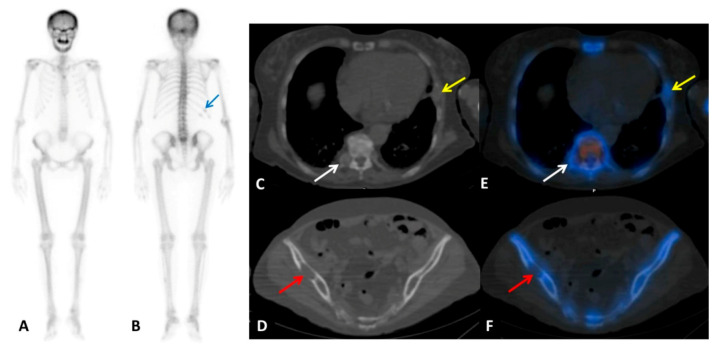
Tc-99m-MDP bone scintigraphy, performed to investigate the unknown bone lesion, showed diffusely increased radiotracer uptake throughout the axial and appendicular skeleton (anterior (**A**) and posterior (**B**) planar views). Especially intense activity is noted in the mandible and maxilla with absent renal and bladder activity, suggesting a “metabolic super-scan” [4]. Of note, the mild uptake in the posterior arch of 11th–12th right ribs (blue arrow) is to be referred to a recent trauma reported in the medical patient history. Additional SPECT/CT acquisition (**C**–**F**) demonstrated no osteo-tropic radiopharmaceutical uptake in the right D5 costovertebral joint (white arrows) and in the left 6th rib (yellow arrows), as well as in the right iliac wing (red arrows) lytic lesions detectable on co-registration CT images.

**Figure 3:**
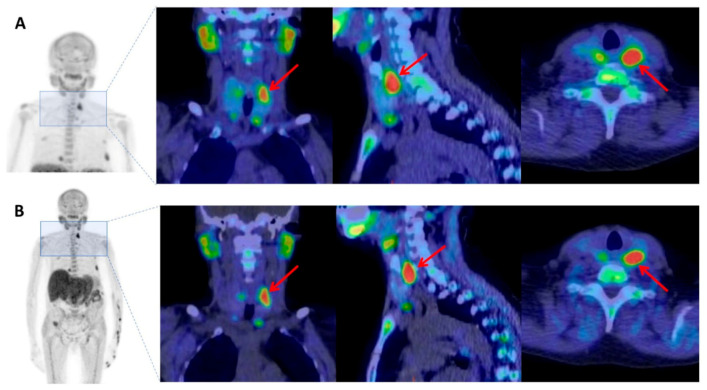
^18^F-FCH PET/CT was performed as the last preoperative imaging. (**A**) A static image acquisition was started 5 min after injection of 2.5 MBq/Kg of ^18^F-FCH from the skull to the diaphragm in order to include any ectopic foci, based on the literature evidence [5,6]. (**B**) A late acquisition at 60 min was subsequently performed, choosing a whole-body protocol because of the evidence of ^18^F-FCH uptake in bones in the early scan. PET/CT clearly showed ^18^F-FCH uptake in the four parathyroid glands, particularly in the left superior one that appeared also enlarged (red arrows).

**Figure 4:**
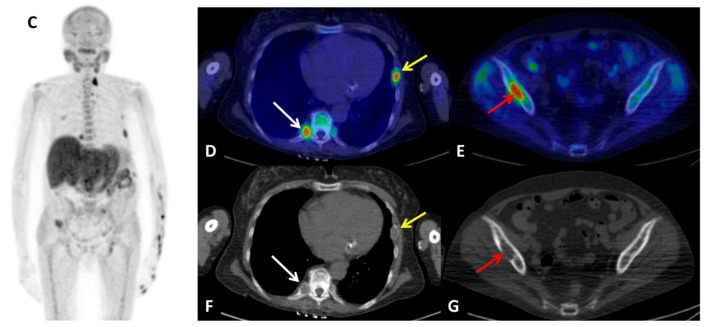
^18^F-FCH PET/CT delayed whole-body acquisition (**C**) demonstrated radiotracer uptake in multiple bone lesions, the most evident in the right D5 costovertebral joint (**D**) (white arrow) and in the left 6th rib (**D**) (yellow arrow), as well as in the right iliac wing (**E**) (red arrow). Their metabolic and morphological (**F**,**G**) aspect were compatible with renal osteodystrophy. A few days later, patient underwent subtotal parathyroidectomy. Three parathyroid glands were removed: two of 20 mm size each at the upper and lower poles of the left thyroid lobe respectively, the third of 15 mm size at the lower pole of the right lobe. The histological examination reported a definitive diagnosis of “parathyroid hyperplasia with chief and oxyphilic cells, nodular pattern”. The day after subtotal parathyroidectomy the biochemical values were: PTH level 16 pg/mL (normal range 15–57 pg/mL), total calcium level of 6.2 mg/dL (normal range 8.5–10.1 mg/dL), ionized calcium level of 2.88 mg/dL (normal range 4.6–5.3 mg/dL). The patient achieved HPT biochemical resolution. The two-week follow-up demonstrated blood values normalization and a further significant reduction of PHT (<6 pg/mL).

In recent years, many studies have been performed on the use of ^18^F-FCH PET/CT in primary hyperparathyroidism with promising results [7,8]. However, its performance in secondary and tertiary hyperparathyroidism is limited [9,10]. Our report showed how nuclear medicine imaging can provide effective and complete diagnostic information, essential for HPT patient management. In particular, ^18^F-FCH PET/CT was demonstrated to be a reliable multimodality imaging technique that, integrating biomolecular and morphologic data, is able to detect with high sensitivity both the hyperfunctioning parathyroid glands and bone involvement, frequently observed in patients with tertiary HPT.

## References

[1] (2009). Special Issue: KDIGO Clinical Practice Guideline for the Care of Kidney Transplant Recipients. Am. J. Transplant..

[2] Jamal S.A., Miller P.D. (2013). Secondary and Tertiary Hyperparathyroidism. J. Clin. Densitom..

[3] Reczek J., Elgazzar A. (2003). Prominent Tc-99m MIBI Skeletal Uptake in Renal Osteodystrophy: A Possible Role for Whole-Body Scanning. Clin. Nucl. Med..

[4] De Graaf P., Schicht I.M., Pauwels E.K.J., te Velde J., de Graeff J. (1978). Bone scintigraphy in renal osteodystrophy. J. Nucl. Med..

[5] Zajíčková K., Zogala D., Kubinyi J. (2018). Parathyroid imaging by 18F-fluorocholine PET/CT in patients with primary hyperparathyroidism and inconclusive conventional methods: Clinico-pathological correlations. Physiol. Res..

[6] Broos W.A.M., Wondergem M., van der Zant F.M., Knol R.J.J. (2019). Dual-time-point 18F-fluorocholine PET/CT in parathyroid imaging. J. Nucl. Med..

[7] Thanseer N., Bhadada S.K., Sood A., Mittal B.R., Behera A., Gorla A.K.R., Kalathoorakathu R.R., Singh P., Dahiya D., Saikia U.N. (2017). Comparative Effectiveness of Ultrasonography, 99mTc-Sestamibi, and 18F-Fluorocholine PET/CT in Detecting Parathyroid Adenomas in Patients With Primary Hyperparathyroidism. Clin. Nucl. Med..

[8] Beheshti M., Hehenwarter L., Paymani Z., Rendl G., Imamovic L., Rettenbacher R., Tsybrovskyy O., Langsteger W., Pirich C. (2018). 18F-Fluorocholine PET/CT in the assessment of primary hyperparathyroidism compared with 99mTc-MIBI or 99mTc-tetrofosmin SPECT/CT: A prospective dual-centre study in 100 patients. Eur. J. Nucl. Med. Mol. Imaging.

[9] Michaud L., Burgess A., Huchet V., Lefèvre M., Tassart M., Ohnona J., Kerrou K., Balogova S., Talbot J.N., Périé S. (2014). Is 18F-fluorocholine-positron emission tomography/computerized tomography a new imaging tool for detecting hyperfunctioning parathyroid glands in primary or secondary hyperparathyroidism?. J. Clin. Endocrinol. Metab..

[10] Broos W.A.M., Wondergem M., Van Der Zant F.M., Knol R.J.J. (2018). Tertiary hyperparathyroidism with renal osteodystrophy on 18F-fluorocholine PET/CT. Clin. Nucl. Med..

